# PPAR*α* Activation Protects against Anti-Thy1 Nephritis by Suppressing Glomerular NF-*κ*B Signaling

**DOI:** 10.1155/2012/976089

**Published:** 2012-05-16

**Authors:** Koji Hashimoto, Yuji Kamijo, Takero Nakajima, Makoto Harada, Makoto Higuchi, Takashi Ehara, Hidekazu Shigematsu, Toshifumi Aoyama

**Affiliations:** ^1^Department of Metabolic Regulation, Institute on Aging and Adaptation, Shinshu University Graduate School of Medicine, 3-1-1 Asahi, Matsumoto 390-8621, Japan; ^2^Department of Nephrology, Shinshu University School of Medicine, 3-1-1 Asahi, Matsumoto 390-8621, Japan; ^3^Department of Pathology, Shinshu University School of Medicine, 3-1-1 Asahi, Matsumoto 390-8621, Japan

## Abstract

The vast increase of chronic kidney disease (CKD) has attracted considerable attention worldwide, and the development of a novel therapeutic option against a representative kidney disease that leads to CKD, mesangial proliferative glomerulonephritis (MsPGN) would be significant. Peroxisome proliferator-activated receptor *α* (PPAR*α*), a member of the steroid/nuclear receptor superfamily, is known to perform various physiological functions. Recently, we reported that PPAR*α* in activated mesangial cells exerted anti-inflammatory effects and that the deficiency of PPAR*α* resulted in high susceptibility to glomerulonephritis. To investigate whether PPAR*α* activation improves the disease activity of MsPGN, we examined the protective effects of a PPAR*α* agonist, clofibrate, in a well-established model of human MsPGN, anti-Thy1 nephritis, for the first time. This study demonstrated that pretreatment with clofibrate (via a 0.02% or 0.1% clofibrate-containing diet) continuously activated the glomerular PPAR*α*, which outweighed the PPAR*α* deterioration associated with the nephritic process. The PPAR*α* activation appeared to suppress the NF-*κ*B signaling pathway in glomeruli by the induction of I*κ*B*α*, resulting in the reduction of proteinuria and the amelioration of the active inflammatory pathologic glomerular changes. These findings suggest the antinephritic potential of PPAR*α*-related medicines against MsPGN. PPAR*α*-related medicines might be useful as a treatment option for CKD.

## 1. Introduction

The vast increase in chronic kidney disease (CKD) has attracted considerable attention worldwide, since CKD is one of the most important risk factors for cardiovascular events, the induction of kidney replacement therapies, and death [1]. Among many types of primary kidney disease, mesangial proliferative glomerulonephritis (MsPGN) including IgA nephropathy is a representative proteinuric kidney disease that leads to CKD [2, 3]. Various medications such as angiotensin-converting enzyme inhibitors, angiotensin II receptor blockers, fish oil, statins, hydroxymethylglutaryl-CoA reductase inhibitors, immunosuppressive therapy, antiplatelets, and anticoagulants have been proposed; however, it remains difficult to control the nephritic activity associated with severe inflammatory pathologic glomerular changes [4]. It is known that the marked activation of nuclear factor kappa B (NF-*κ*B) was detected in various kidney cells from MsPGN patients, including mesangial cells, glomerular endothelial and epithelial cells, tubular epithelial cells, and infiltrating cells and that the NF-*κ*B transcriptional activation is significantly involved in the progression of kidney tissue injury [5]. Therefore, the development of a novel therapeutic option against NF-*κ*B activation in active MsPGN would be significant.

Peroxisome proliferator-activated receptor *α* (PPAR*α*), a member of the steroid/nuclear receptor superfamily of ligand-dependent transcription factors, is known to perform various physiological functions, including the maintenance of lipid and glucose homeostasis, the regulation of cell proliferation, and anti-inflammatory effects via suppression of the NF-*κ*B pathway [6–13]. Recently, we reported that the activated mesangial cells expressed a significant amount of PPAR*α* and that the representative PPAR*α* agonists, fibrates, exert anti-inflammatory effects in an *in vitro* study using murine mesangial cells stimulated by lipopolysaccharide [14]. Moreover, we also showed that a deficiency of PPAR*α* resulted in high susceptibility to glomerulonephritis in an *in vivo* murine study [15]. These findings suggest that glomerular PPAR*α* activation might contribute to the treatment of MsPGN.

To obtain basic evidence concerning the beneficial potential of PPAR*α* ligand against MsPGN, we examined the glomerular protective effects of a PPAR*α* agonist, clofibrate, in a well-established rat model of human MsPGN, anti-Thy1 nephritis. Anti-Thy1 nephritis, induced by anti-Thy1 antibody binding to the corresponding antigen on the membrane of mesangial cells, is marked by obvious transient inflammatory glomerular lesions, such as mesangial cell proliferation, mesangiolysis, glomerular capillary aneurysm formation, and extracapillary proliferation [16]. Several earlier studies demonstrated that upregulation of the NF-*κ*B gene was greatly involved in the developmental process of anti-Thy1 nephritis [17, 18]. The current study reveals for the first time that PPAR*α* activation via clofibrate treatment would attenuate the disease activity of anti-Thy1 nephritis by suppressing glomerular NF-*κ*B signaling.

## 2. Materials and Methods

### 2.1. Animals and Experimental Design

Male Wistar rats were used in this study (age, 8 weeks; purchased from Nihon SLC, Hamamatsu, Japan). All rats were maintained in a facility free of specific pathogens, housed in a temperature- and light-controlled environment (25°C; 12-h light/dark cycle), and given tap water *ad libitum*. All procedures were performed in accordance with the guidelines of the Shinshu University, the National Institutes of Health, and the Association for Assessment and Accreditation of Laboratory Animal Care. The rats were divided into three groups: a regular diet group (Fib(−); *n* = 24), a low-dose clofibrate-containing-diet group (0.02% Fib; *n* = 12), and a high-dose clofibrate-containing-diet group (0.1% Fib; *n* = 24). The rats in the Fib(−) group were fed a regular diet throughout the experimental period. The clofibrate-treated rats were fed a 0.02 or 0.1% clofibrate-containing diet (drug weight/food weight) beginning 5 days before the injection of anti-Thy1 antibody, respectively. We measured the animals' body weight and daily food consumption every day. The mean body weight and food consumption values in each group did not change significantly throughout the experimental period, and did not differ among groups (Table 1). Using these data, the mean ± SD clofibrate dosage was calculated (Table 1). Clofibrate was obtained from Wako (Tokyo, Japan). Anti-Thy1 MsPGN was induced by a single intravenous injection of a mouse anti-Thy1 monoclonal antibody-containing solution. Concentrated anti-Thy1 antibody solution was obtained from Cedarlane Laboratories (Ontario, Canada, catalog no. CL005A). One vial of the commercial antibody solution was diluted with 300 *μ*L of sterile saline, and it was injected into each rat at a dose of 25 *μ*L/100 g body weight. No rat in any group died except those sacrificed according to the study protocol throughout the experimental period. Some rats were sacrificed for analysis according to the protocol at days 0, 4, 7, and 14. The numbers of rats subjected to analyses at days 0, 4, 7, and 14 were as follows: Fib(−) group, *n* = 6; 0.02% group, *n* = 3; 0.1% group, *n* = 6, at each day, respectively. The possibility of the induction failure of nephritis was checked by means of the measurement of urine protein excretion in the early phase, as described below. In the current study, all rats, which were injected to anti-Thy1 antibody solution, developed significant increases of proteinuria at day 2, indicating perfect induction of anti-Thy1 nephritis.

### 2.2. Pathological Analyses

Tissues from the kidneys of rats in each group were fixed in 4% paraformaldehyde. Deparaffinized sections were stained with hematoxylin & eosin, periodic acid Schiff, or periodic acid-methenamine-silver. Since anti-Thy1 nephritis markedly caused various acute inflammatory glomerular changes including mesangial cell proliferation, mesangiolysis, glomerular capillary aneurysm formation, and extracapillary proliferation, we evaluated these inflammatory glomerular changes using semiquantitative pathologic analyses. For the analyses, 50 randomly selected glomeruli from each kidney section were studied. The degree of mesangial cell proliferation was estimated using a scale that ranged from 0 to 3 (0, normal; 1, mild; 2, moderate; 3, severe). Indices were calculated using the following formula: Index = (*n*_0_ × 0)+(*n*_1_ × 1)+(*n*_2_ × 2)+(*n*_3_ × 3)/50  (∑_*n*_ = 50). The levels of severity of the mesangiolysis, glomerular capillary aneurysm formation, and crescent formation were assessed by the appearance rate of each finding (% of the damaged glomeruli). These pathologic analyses were performed in a blinded manner by two observers who were unaware of the study protocol.

### 2.3. Intranuclear Transcription Factor Assay

The specific transcription factor DNA-binding activities of PPAR*α* or NF-*κ*B in nuclear extracts were analyzed using enzyme-linked immunosorbent assay (ELISA) kits (Cayman Chemical, CA, USA). The specific double-stranded DNA sequence containing the PPAR or NF-*κ*B response element was immobilized onto the bottoms of the wells of a 96-well plate. PPAR*α* or NF-*κ*B, contained in a nuclear extract, bound to each specific response element and was detected by the addition of a specific primary antibody. After secondary antibody binding, the DNA-binding activity was visualized calorimetrically. These ELISA assays are nonradioactive, sensitive established methods, and recently replaced the radioactive electrophoretic mobility shift assay. Nuclear protein was extracted from isolated glomeruli using the NE-PER Nuclear and Cytoplasmic Extraction Kit (Thermo Scientific, MA, USA). Glomeruli were isolated from the kidney cortex of each rat by mechanical sieving techniques as described previously [19]. The nuclear protein samples, as well as commercial positive control protein reagents and blank samples, were subjected to ELISA in triplicate. The mean optical density (OD) of the blank sample was subtracted from the OD of each sample, and the value was normalized to each nuclear protein amount and subsequently expressed as the change relative to the value of the control rats (Fib(−) group at day 0).

### 2.4. Analyses of mRNA

Analyses of mRNA were performed using quantitative real-time PCR as described previously [20–22]. One microgram of total RNA, extracted from isolated glomeruli obtained from each rat, was reverse-transcribed using oligo(dT) primers and Superscript reverse transcriptase (Invitrogen, CA). The cDNAs were quantified with an ABI PRISM 7700 sequence detection system (Applied Biosystems, CA) using specific primers and SYBR Green double-stranded DNA binding dye I. The specific primers were designed as shown in Table 2. For relative quantification of mRNA, glyceraldehyde-3-phosphate dehydrogenase was used as an internal control, and the relative expression of RNA was calculated by the comparative threshold cycle (Ct) method. The expression was expressed in terms of the change relative to the expression of the control [Fib(−) group of rats at day 0]. PCR reactions were carried out in triplicate and averaged for analysis.

### 2.5. Miscellaneous Methods

Throughout the experimental period, urine collections were carried out daily. Urine protein concentrations were measured as described previously [7]. Serum urea nitrogen and serum creatinine were measured by enzymatic methods using a clinical analyzer (JCA-BM2250; JEOL, Tokyo, Japan).

### 2.6. Statistical Analysis

Analysis of significant differences with respect to the interactive effects of the two factors (fibrate treatment and anti-Thy1 antibody injection) was performed using one-way ANOVA. Throughout the paper, significant differences from the respective day 0 group are indicated with number signs (^#^ *P* < 0.05, ^##^ *P* < 0.01, ^###^ *P* < 0.001), while significant differences between regular-diet and clofibrate-diet groups are indicated with asterisks (*P < 0.05, **P < 0.01, ***P < 0.001).

## 3. Results

### 3.1. The Antiproteinuric Effect by Clofibrate Treatment in Anti-Thy1 Nephritis

**Figure 1:**
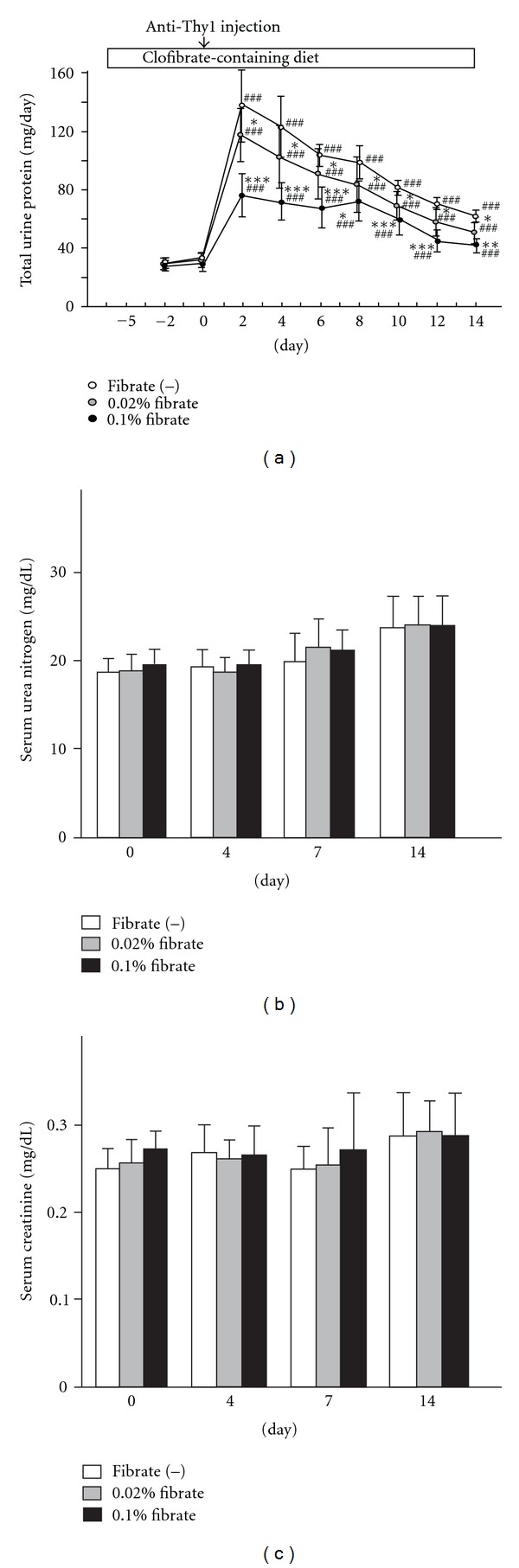
Alteration of kidney function by clofibrate pretreatment in anti-Thy1 nephritis rat. (a) Time course of the daily urinary protein excretion in anti-Thy1 nephritis rats. ((b) and (c)) Serum concentrations of urea nitrogen and creatinine, respectively. The clofibrate-pretreatment group was fed a 0.02% or 0.1% clofibrate-containing diet from 5 days before anti-Thy1 antibody injection. The start time of the anti-Thy1 antibody injection was designated as day 0. Values represent means ± SD (*n* = 24, 12, and 24 for the Fib(−), 0.02% fibrate, and 0.1% fibrate groups, resp.). Significant differences from the respective day 0 groups are indicated with number signs ^(###^ *P* < 0.001), while significant differences between regular-diet and clofibrate-diet groups are indicated with asterisks (*P < 0.05, **P < 0.01, ***P < 0.001).

Pretreatment with clofibrate for 5 days and the inductive procedure of anti-Thy1 nephritis did not cause any systemic changes to body weight, food consumption, urine volume, blood pressure, or heart rate (Table 1). Pretreatment with clofibrate did not affect the urinalysis in any group of rats; however, the anti-Thy1 antibody injection immediately and dramatically increased daily urine protein excretion in all groups (Figure 1). Especially in the Fib(−) group, massive proteinuria appeared within 2 days and then gradually decreased. The clofibrate treatment attenuated the marked elevation of proteinuria throughout the experimental period in a dose-dependent manner. Serum levels of urea nitrogen and creatinine were prone to increase in all groups; however, there were no significant differences among the three groups. These findings suggest an antiproteinuric effect of clofibrate treatment against anti-Thy1 nephritis.

### 3.2. The Amelioration of Glomerular Active Lesions by Clofibrate Treatment

**Figure 2:**
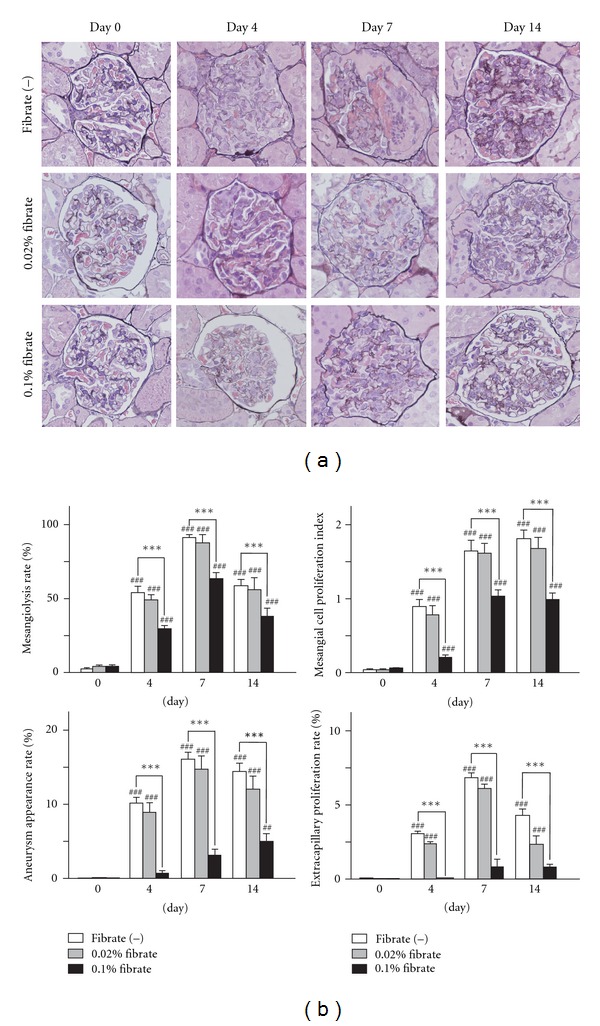
Light microscopic analyses of kidney injuries. (a) Representative micrographs of the kidney in anti-Thy1 nephritis rats. Kidney sections were stained with periodic acid methenamine silver (PAM). (b) Semiquantification of pathologic changes including mesangial cell proliferation, mesangiolysis, glomerular capillary aneurysm formation, and crescent formation. Values are means ± SD. Significant differences from the respective day 0 groups are indicated with number signs (^##^ *P* < 0.01, ^###^ *P* < 0.001), while significant differences between regular-diet and clofibrate-diet groups are indicated with asterisks (***P < 0.001).

To evaluate kidney damage, we carried out pathological analyses. In the Fib(−) group, an acute finding of mesangial damage, mesangiolysis, induced by anti-Thy1 antibody appeared within 4 days, followed by various severe glomerular inflammatory changes, such as glomerular capillary aneurysm formation, crescent formation, and mesangial cell proliferation (Figure 2). The semiquantitative pathological analyses demonstrated that the levels of severity of these acute glomerular lesions reached peak levels on day 7 in the Fib(−) group. The findings of mesangiolysis, capillary aneurysm, and crescent improved spontaneously on day 14, while the high level of mesangial cell proliferation continued in this group. The pretreatment with clofibrate caused no glomerular change at day 0. This treatment markedly moderated the acute findings induced by anti-Thy1 antibody in a dose-dependent manner throughout the experimental period. These findings suggest that pretreatment with clofibrate ameliorated the glomerular active lesions of anti-Thy1 nephritis.

### 3.3. The Activation of Glomerular PPAR*α* by Clofibrate Treatment

**Figure 3:**
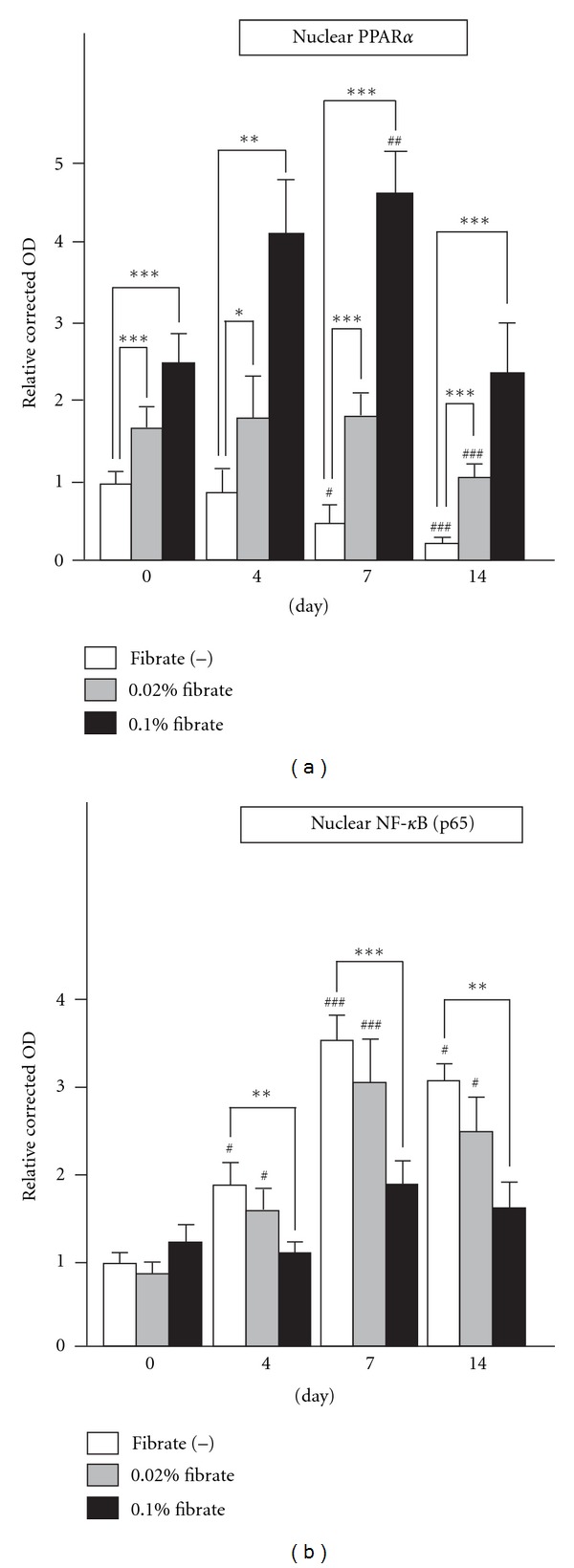
Alteration of intranuclear transcription factor activities. (a) The PPAR response element-binding activities of intranuclear PPAR*α* from glomeruli in each group of anti-Thy1 nephritis rats. (b) The NF-*κ*B response element-binding activities of intranuclear NF-*κ*B in each group. For these assays, the nuclear protein samples were subjected to ELISA in triplicate. The optical density (OD) for each sample was corrected by that of a blank sample and by protein amount in each sample. The data were expressed as changes relative to the value for the control rats (Fib(−) group of rats at day 0). Values are means ± SD. Significant differences from the respective day 0 groups are indicated with number signs (^#^ *P* < 0.05, ^##^ *P* < 0.01, ^###^ *P* < 0.001), while significant differences between regular-diet and clofibrate-diet groups are indicated with asterisks (*P < 0.05, **P < 0.01, ***P < 0.001).

**Figure 4:**
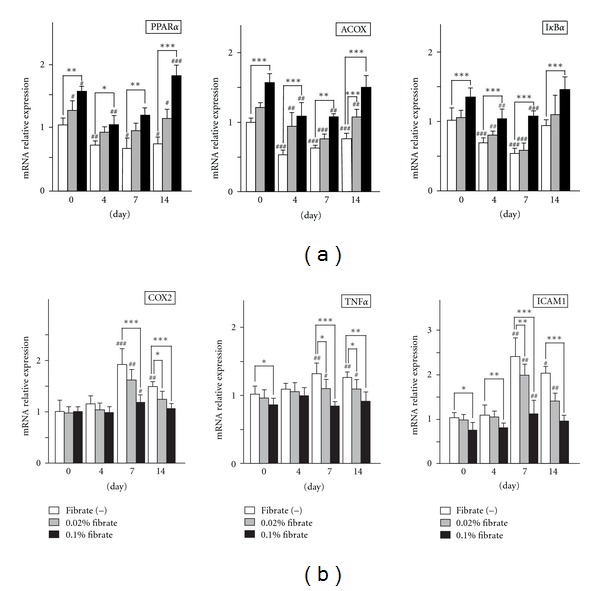
Alteration of mRNA expression of target molecules of transcription factors. mRNAs were obtained from glomeruli of each group of rats. The expression of mRNAs for the target molecules of PPAR*α* (a) and NF-*κ*B (b), including PPAR*α*, ACOX, I*κ*B*α*, COX2, TNF*α*, and ICAM1, was measured with real-time PCR. For relative quantification of mRNA, glyceraldehyde-3-phosphate dehydrogenase was used as an internal control, and the relative expression of RNA was calculated by the comparative threshold cycle (Ct) method. The expression was expressed as the change relative to that of the control rats (Fib(−) group of rats at day 0). PCR reactions were carried out in triplicate and averaged for analysis. Values represent means ± SD. Significant differences from the respective day 0 groups are indicated with number signs (^#^ *P* < 0.05, ^##^ *P* < 0.01, ^###^ *P* < 0.001), while significant differences between regular-diet and clofibrate-diet groups are indicated with asterisks (*P < 0.05, **P < 0.01, ***P < 0.001).

To investigate the degree of PPAR*α* activation via clofibrate treatment, we examined the binding activities of intranuclear PPAR*α* with PPAR response element (PPRE), using nuclear protein samples from isolated glomeruli of each group. In the Fib(−) group, the induction of anti-Thy1 nephritis obviously decreased the PPRE binding activity of PPAR*α* at days 7 and 14 in a time-dependent manner (Figure 3). The pretreatment with clofibrate increased the glomerular PPAR*α* activity at day 0 (before the anti-Thy1 antibody injection), in a dose-dependent manner. In spite of the induction of anti-Thy1 nephritis, the high-dose clofibrate treatment further enhanced the increase of PPAR*α* activity, and the low-dose treatment maintained the activated level as of day 7. Then, the PPAR*α* activities of both clofibrate groups decreased at day 14, but the level of activity was still high as compared to that of the control rats. These findings suggest that the PPRE binding activity of PPAR*α* deteriorated due to the development of anti-Thy1 nephritis in the control group; however, the pretreatment with clofibrate outweighed this deterioration and continuously activated glomerular PPAR*α*. To verify the enhancement of the transcriptional activity of glomerular PPAR*α*, we next examined the mRNA expression levels of PPAR*α* and of its representative target molecule, acyl-CoA oxidase (ACOX). In the Fib(−) group, the induction of anti-Thy1 nephritis decreased the mRNA expressions of PPAR*α* and ACOX, a result that was identical to the results of the PPRE binding assay and suggesting the deterioration of glomerular PPAR*α* (Figure 4). The pretreatment with clofibrate increased the mRNA expression of PPAR*α* and ACOX at day 0 in a dose-dependent manner. The induction of anti-Thy1 nephritis decreased these expressions in each group; however, the PPAR*α* and ACOX expressions of both clofibrate treatment groups remained over the baseline level of the control rats. These findings support the finding of continuous activation of PPAR*α* via the clofibrate treatment. This activation was resistant to the PPAR*α* deterioration associated with the nephritic process.

### 3.4. The Suppression of the NF-*κ*B Pathway by Clofibrate Treatment

Since many earlier studies have demonstrated that the activated PPAR*α* exerts anti-inflammatory effects through suppression of the NF-*κ*B pathway [23, 24], we next examined the binding activities of nuclear NF-*κ*B (p65) with a NF-*κ*B response element. The response element binding activities of NF-*κ*B did not differ among the groups at day 0. However, anti-Thy1 antibody injection increased the NF-*κ*B binding activities in the Fib(−) and low-dose clofibrate groups (Figure 3). The peak phase of NF-*κ*B activation in both groups appeared to be around day 7. On the other hand, the high-dose clofibrate treatment dramatically suppressed the NF-*κ*B activation throughout the experimental period. The time course of NF-*κ*B activation appeared to be consistent with that of the pathological activities of anti-Thy1 nephritis in each group. It is known that activated PPAR*α* suppresses the NF-*κ*B pathway via the induction of the inhibitory factor *κ*B*α* (I*κ*B*α*) [23]. The mRNA analyses demonstrated that the high-dose clofibrate treatment continuously induced I*κ*B*α* expression and decreased the high mRNA levels of proinflammatory mediators including cyclooxygenase-2 (COX2), tumor necrosis factor-*α* (TNF*α*), and intercellular adhesion molecule-I (ICAM1), which were NF-*κ*B target molecules (Figure 4). These findings suggest that the anti-Thy1 antibody injection induced the continuous activation of the NF-*κ*B signaling pathway in glomeruli followed by an increase of proinflammatory mediators and that this proinflammatory pathway was suppressed considerably by the induction of I*κ*B*α*, which might be mediated by PPAR*α* activation.

## 4. Discussion

The current study demonstrated that pretreatment with clofibrate exerted antiproteinuric effects and ameliorated the active glomerular pathologic inflammatory changes in rat anti-Thy1 nephritis. The pretreatment with clofibrate continuously activated the glomerular PPAR*α*, which outweighed the PPAR*α* deterioration associated with the nephritic process. This glomerular PPAR*α* activation would suppress the NF-*κ*B signaling pathway via the induction of I*κ*B*α* and result in beneficial antinephritic effects. These findings indicate the anti-nephritic potentiality of PPAR*α*-related medicines.

Several metabolic experimental studies, including murine studies employing a high-fat-diet-induced glomerular injury model or a diabetic nephropathy model, have also demonstrated the beneficial properties of the PPAR*α* agonist fibrates in reducing glomerular lesions [25–28]. These studies suggested various beneficial glomerular protective effects of fibrates as follows. First, PPAR*α* activation improves the lipid metabolic abnormality in glomeruli. Second, PPAR*α* activation attenuates the glomerular oxidative stress. Third, PPAR*α* activation ameliorates systemic insulin resistance, lipid abnormality, energy homeostasis, hypertension, and vascular injuries. These pathogenic abnormalities are known to induce secondary activation of the NF-*κ*B signaling pathway and accumulation of the extracellular matrix in glomeruli, resulting in glomerular failure [25]. Using these metabolic experimental models, it might be difficult to detect whether PPAR*α* agonists have direct anti-inflammatory effects that protect glomeruli. In contrast to these metabolic models, the mechanism of glomerular injury of anti-Thy1 nephritis is due to the direct inflammatory response by complement (C5b-9)-induced activation of the NF-*κ*B signaling pathway [16, 17]. Therefore, this animal model appeared to be suitable to demonstrate the direct anti-inflammatory effects of PPAR*α* in glomerulonephritis. Another earlier experimental study using a rat antiglomerular basement membrane crescentic glomerulonephritis model also indicated the direct anti-inflammatory effects of PPAR*α*, thus supporting our results [29]. The NF-*κ*B-suppressing effects of glomerular PPAR*α* might be useful for the treatment of the various types of glomerulonephritis, including MsPGN, immune complex kidney disease, crescentic glomerulonephritis, and lupus nephritis, as well as metabolic abnormality-based glomerulonephropathy.

In the current study, the NF-*κ*B-suppressing effects of clofibrate might be obscure in the low-dose fibrate treatment group; however, the antiproteinuric effect in this group was rather obvious, suggesting the existence of another mechanism of the anti-proteinuric effect of PPAR*α* agonist. A recent study reported that a PPAR*α* agonist, fenofibrate, effectively reduced proteinuria and attenuated the reduction level of glomerular nephrin, an important molecule regulating glomerular permeability, following doxorubicin-induced podocyte injuries [30]. This study also demonstrated that PPAR*α*-null mice exhibited susceptibility to doxorubicin-induced proteinuria, which was associated with lower expression of nephrin compared with wild-type mice. This paper suggests the existence of an anti-proteinuric effect of PPAR*α* agonist via the maintenance effect of nephrin. Several previous studies reported that the nephrin protein expression in the glomeruli of anti-Thy1 nephritis was weak and exhibited a discontinuous pattern as determined by immunostaining [31]. Therefore, the anti-proteinuric effects of PPAR*α* agonists might be derived from this protective effect of podocytes, as well as from NF-*κ*B-suppressing effects.

It is known that PPAR*α* is expressed more highly in proximal tubular epithelial cells (PTECs) than in glomeruli, and tubular PPAR*α* exerts a protective effect in PTECs via the amelioration of fatty acid catabolism, the decreasing of oxidative stress and apoptosis, and the suppression of NF-*κ*B singling [13, 32]. These tubular protective effects of PPAR*α* were detected in various types of tubulointerstitial injury models, such as protein-overload nephropathy (the toxicity of excess fatty acids), unilateral ureteral obstruction, 5/6 nephrectomy, ischemia/reperfusion injury, and cisplatin injury [13, 33–35]. In the current study, anti-Thy1 nephritis induced a high level of proteinuria, a representative tubulotoxic factor; however, the proteinuria was transient, so tubulointerstitial changes were scarcely detected throughout the experimental period. Therefore, we could not detect the protective effects of PPAR*α* against tubulointerstitial injuries derived from a high level of proteinuria in this model. In order to detect such effects, we would have to perform another experiment using models exhibiting continuous excretion of proteinuria in the future.

In the current study, we used clofibrate to investigate the antinephritic potential of PPAR*α*-related medicines, since this molecule was established as a representative beneficial medicine activating PPAR*α*. However, we recommend the careful use of fibrates when clinical physicians treat kidney disease patients because the renal toxicity by excess serum accumulation of the fibrates was often detected in the animal models of kidney dysfunction [36]. The mechanism of the renal toxicity of fibrates was not fully understood; however, our earlier studies reported that excess-dose clofibrate treatment induced considerable oxidative stress due to a PPAR*α*-dependent mechanism, such as the induction of PPAR*α*-regulated ROS-generating enzymes (acyl-CoA oxidase, cytochrome P450 4A, and NADPH oxidase) and the enhancement of mitochondrial fatty acid *β*-oxidation [13, 37]. Furthermore, our recent study reported that fibrates could also enhance oxidative stress in a PPAR*α*-independent manner, as well as by a PPAR*α*-dependent mechanism [36]. The unfavorable oxidative effect of fibrates treatment appeared to surpass the antioxidative effects of PPAR*α* activation in the situation of excess drug accumulation in the serum; therefore adequate dose management would be essential for patients with kidney dysfunction. The earlier study demonstrated that the marked elevation of oxidative stress, induced by excess serum accumulation of fibrates, exerted proximal tubular epithelial cell toxicities such as tubular dilatation, tubular atrophy, and tubular cast formation [36]. Interestingly, the glomerular toxicity of fibrates was not detected, suggesting that excess fibrates exert only tubular toxicity without glomerular toxicity [36]. In the current study, the anti-Thy1 nephritic process resulted in transient glomerular damage without tubular damage; therefore, this limited situation might contribute to the good results of fibrate effects, obvious glomerular protection, and less tubular toxicity. On the other hand, in the human case of many types of chronic progressing glomerulonephritis, a considerable level of gradual secondary tubular damage generally appears; therefore, the tubular toxic effects of fibrates might become obvious, especially after CKD has progressed. With regard to the safe use of fibrates, we must be clear that the results of this study would not provide long-term safety verification for CKD patients. Furthermore, in order to prevent excess drug accumulation and the associated toxicities, we employed a pretreatment protocol established via past animal study [36] in which an adequate dose of fibrate was started before the appearance of apparent kidney dysfunction. In these specific situations, we succeeded in detecting beneficial anti-nephritic effects of fibrates without adverse renal effects in the current study. We believe that the results are important when considering the beneficial potential of PPAR*α*-related medicine in treating glomerulonephritis. In humans, two clinical trials have reported that fibrates suppressed microalbuminuria in patients with early diabetic nephropathy; however, kidney dysfunction was not obviously improved [38, 39]. The results of these clinical trials might be derived from the delicate balance between the beneficial effects of PPAR*α* activation and the renal toxicity of fibrates. In the future, the development of a novel PPAR*α* agonist exhibiting stable pharmacokinetics under kidney dysfunction is needed.

## 5. Conclusions

Taken together, the current results suggest that pretreatment with a representative PPAR*α* agonist, clofibrate, exerts a protective function against anti-Thy1 nephritis via the suppression of glomerular NF-*κ*B signaling for the first time. The developmental process of anti-Thy1 nephritis decreased glomerular PPAR*α* expression and weakened its function, while the pretreatment with an appropriate dose of clofibrate appeared to outweigh this deterioration. However, there are several limitations to our study. First, the use of pretreatment before nephritis might not fit the actual clinical situation of the treatment for human kidney disease. The investigation of the beneficial effects of a treatment administered after the initiation of anti-Thy1 nephritis using a novel medicine, a high serum concentration of which is not caused by kidney dysfunction or scarcely exerts toxicity, is needed in the future. Second, there are known to be species differences in PPAR*α* activation via fibrate treatment between rodents and humans [40]. Therefore, we could not directly apply the results of the current study to human patients. In order to evaluate the anti-nephritic effect of human PPAR*α* function, an investigation using PPAR*α*-humanized mice might be useful [41]. Third, anti-Thy1 nephritis is a very famous rat model resembling human MsPGN; however this nephritis could be produced only in rats. Therefore, we have to verify the anti-nephritic effects of PPAR*α* agonists using various nephritic models in the future. Nevertheless, the potential anti-nephritic effects of PPAR*α* activation suggested in the current study will be valuable for the development of a useful therapeutic strategy to treat glomerulonephritis.

## Figures and Tables

**Table 1:** Systemic change and estimated dose of clofibrate in anti-Thy1 nephritis rats.

Parameter	Fibrate (−) group				0.02% fibrate group				0.1% fibrate group			
	Day 0	Day 4	Day 7	Day 14	Day 0	Day 4	Day 7	Day 14	Day 0	Day 4	Day 7	Day 14
BW (g)	248 ± 26	254 ± 33	256 ± 38	267 ± 54	257±16	259 ± 48	264 ± 35	265 ± 47	254 ± 21	258 ± 39	263 ± 30	267 ± 40
sBP (mmHg)	152 ± 17	158 ± 20	158 ± 15	150±10	155 ± 10	149 ± 12	152 ± 18	153 ± 6	151 ± 16	157 ± 15	155 ± 20	158 ± 15
HR (beat/min)	400 ± 16	407 ± 30	410 ± 20	400 ± 20	393 ± 11	400 ± 10	406 ± 21	404±25	396 ± 26	405 ± 36	403 ± 30	402±15
FC (g/day)	20.1 ± 5	19 ± 3	18.5 ± 5	18.3 ± 5	18.2 ± 2	18.7 ± 2	19.6 ± 2	18.6 ± 5	18.9 ± 3	18.7 ± 1	19.5 ± 3	19 ± 6
Clo dose (mg/kg)	0	0	0	0	14.2	14.1	14.8	14.1	74.5	72.4	74.1	71.2

BW, body weight; sBP, systolic bood pressure; HR, heart rate; FC, food consumption; Clo, clofibrate.

These parameters were not affected by the induction of anti-Thy1 nephritis. There was no significant difference among groups.

**Table 2:** Primer sequences for quantitative real-time PCR assay.

Gene name	Primers	GenBank access no.
PPAR*α*	Forward: 5′-GACAAGGCTCAGGATACCACTATG-3′ Reverse: 5′-TTGCAGCTTCGATCACACTTGTC-3′	NM_013196
ACOX	Forward: 5′-GGGCCTGACAGAAGCCTACAAG-3′ Reverse: 5′-AAGGTCGACAGAGGTTAGGTTCCA-3′	NM_017340
I*κ*B*α*	Forward: 5′-TGACCATGGAAGTGATTGGTCAG-3′ Reverse: 5′-GATCACAGCCAAGTGGAGTGGA-3′	NM_001105720
COX2	Forward: 5′-GCGACTGTTCCAAACCAGCA-3′ Reverse: 5′-TGGGTCGAACTTGAGTTTGAAGTG-3′	NM_017232
ICAM1	Forward: 5′-ACAAGTGCCGTGCCTTTAGCTC-3′ Reverse: 5′-GATCACGAAGCCCGCAATG-3′	NM_012967
TNF*α*	Forward: 5′-AACTCGAGTGACAAGCCCGTAG-3′ Reverse: 5′-GTACCACCAGTTGGTTGTCTTTGA-3′	NM_012675

PPAR*α*, peroxisome proliferator-activated receptor *α*; ACOX, acyl-CoA oxidase; I*κ*B*α*, inhibitory factor *κ*B*α*; COX2, cyclooxygenase-2; ICAM1, intercellular adhesion molecule-1; TNF*α*, tumor necrosis factor-*α*.
